# Associations between the pupil light reflex and the broader autism phenotype in children and adults

**DOI:** 10.3389/fnhum.2022.1052604

**Published:** 2023-02-21

**Authors:** Sapir Soker-Elimaliah, Aviva Lehrfield, Samuel R. Scarano, Jennifer B. Wagner

**Affiliations:** ^1^Department of Psychology, College of Staten Island, City University of New York, New York, NY, United States; ^2^Department of Psychology, The Graduate Center, City University of New York, New York, NY, United States; ^3^Mrs. T.H. Chan Division of Occupational Science and Occupational Therapy, University of Southern California, Los Angeles, CA, United States; ^4^Albert Einstein College of Medicine, The Bronx, NY, United States

**Keywords:** pupil light reflex, pupillometry, sensory sensitivity, broader autism phenotype, restricted and repetitive behaviors

## Abstract

The pupil light reflex (PLR), a marker of neuronal response to light, is a well-studied index of autonomic functioning. Studies have found that autistic children and adults have slower and weaker PLR responses compared to non-autistic peers, suggesting lower autonomic control. Altered autonomic control has also been associated with increased sensory difficulties in autistic children. With autistic traits varying in the general population, recent studies have begun to examine similar questions in non-autistic individuals. The current study looked at the PLR in relation to individual differences in autistic traits in non-autistic children and adults, asking how differences in the PLR could lead to variation in autistic traits, and how this might change across development. Children and adults completed a PLR task as a measure of sensitivity to light and autonomic response. Results showed that, in adults, increased levels of restricted and repetitive behaviors (RRB) were associated with a weaker and slower PLR. However, in children, PLR responses were not associated with autistic traits. Differences in PLR were also found across age groups, with adults showing smaller baseline pupil diameter and stronger PLR constriction as compared with children. The current study expanded on past work to examine the PLR and autistic traits in non-autistic children and adults, and the relevance of these findings to sensory processing difficulties is discussed. Future studies should continue to examine the neural pathways that might underlie the links between sensory processing and challenging behaviors.

## Introduction

Autism spectrum disorder is a neurodevelopmental condition characterized by social interaction and communication difficulties and restricted interests and repetitive behaviors (RRB). RRB can be displayed by stereotyped or repetitive motor behaviors, focused areas of interest, insistence on sameness, and by hyper- or hypo-responsivity to sensory input (American Psychiatric Association, 2013).

A growing body of research has asked what biological mechanisms might underlie the difficulties seen in autistic individuals, including differences in cerebral activity (e.g., Eack et al., 2017; Wolff et al., 2017; Abbott et al., 2018; Jung et al., 2019; McKinnon et al., 2019; Sato and Uono, 2019; Ecker et al., 2022) and genetic factors (e.g., Cantor et al., 2018; Ramaswami and Geschwind, 2018; Waye and Cheng, 2018; Wiśniowiecka-Kowalnik and Nowakowska, 2019; Yousaf et al., 2020; Warrier et al., 2022). Another potential factor that has been examined is the autonomic nervous system (ANS), which regulates involuntary processes in the human body, such as breathing and heart rate (e.g., Iaizzo and Fitzgerald, 2015). The ANS includes two primary branches, the sympathetic nervous system (SNS) and the parasympathetic nervous system (PNS), which work cooperatively to regulate internal processes according to conditions both inside and outside of the body. The SNS prepares the body for intense physical activity as a response to a stressful event (“fight or flight” responses), while the PNS helps to maintain homeostasis during periods of rest and recuperation (“rest and digest” responses).

One common measure used to study autonomic activity is pupillometry, which assesses pupil diameter at baseline or in response to a stimulus (Beatty and Lucero-Wagoner, 2000). The primary factor that influences pupil diameter is changes in illumination, and pupil constriction or dilation are directly linked to the amount of light entering the eye. Pupil responses can reflect the interaction and balance between the sympathetic and parasympathetic branches working together to regulate pupil size at any given time (Goldwater, 1972). For example, an increase in pupil diameter, or pupil dilation, can be a result of either an increase in SNS activity or a decrease in PNS activity (Steinhauer et al., 2004). Therefore, measures of pupillary responses often indicate general autonomic activity.

Researchers have discussed indicators of subcortical activity in relation to pupillary responses (e.g., Bast et al., 2018, 2021). For example, studies have linked arousal levels, as observed by pupil constriction and dilation, to brain activity through two paths. One suggested path to changes in pupil diameter goes through the locus coeruleus and links arousal levels with cognitive and behavioral flexibility (for a review, see Poe et al., 2020). A second path goes through the superior colliculus, which is linked to attention shifting and regulating stress-induced responses, and can also underlie cognition- and behavior-related changes in pupil diameter (for a review, see Wang and Munoz, 2015). Both paths are related to activation of the PNS and SNS (Hall and Chilcott, 2018).

The pupil light reflex (PLR), which refers to changes in pupil diameter in response to a quick flash of light, is a reliable marker of autonomic function that is regularly used in clinical settings to assess neurological processes (e.g., Cocker et al., 2005), including intensive care units (e.g., Bower et al., 2021). In addition to clinical settings, the PLR is also used in non-clinical research settings (e.g., Bremner, 1999; Beatty and Lucero-Wagoner, 2000). PLR responses have been described in terms of three phases, with the initial phase of rapid constriction in response to light controlled primarily by PNS activity, the second phase characterized by a rapid dilation controlled by both the PNS and the SNS, and the third phase characterized by a slower dilation that is mainly controlled by the SNS (e.g., Wang et al., 2016). Reduced PNS responding was found to correspond to a less robust PLR in this first phase, including smaller constriction amplitude and slower latency to constrict (Levy et al., 1992; Wang et al., 2016).

Various aspects of pupillometry have been studied in autistic individuals. Measures of the initial phase of the PLR have been consistently found to differ between autistic and non-autistic individuals across numerous studies, with slower and less pronounced PLR in autism (e.g., Fan et al., 2009; Daluwatte et al., 2013, 2015; Dinalankara et al., 2017; Lynch, 2018), suggesting reduced parasympathetic activity. These diminished PLR responses were found to also correlate with more sensory processing difficulties in autistic children (Daluwatte et al., 2015). Interestingly, infants at increased likelihood for autism (by virtue of an older autistic sibling) show a *stronger* PLR response by the age of 10 months (Nyström et al., 2015), and stronger PLR responses predicted greater autism symptomology at age 3 years (Nyström et al., 2018), suggesting changes in how the PLR might relate to autism and autistic traits across development.

Results with other pupillary measures have been mixed. For example, while some studies report differences between autistic and non-autistic individuals in both baseline pupil diameter (e.g., Anderson and Colombo, 2009; Martineau et al., 2011) and task-related pupil responses (e.g., Falck-Ytter, 2008; Blaser et al., 2014; Polzer et al., 2022), other studies have found no differences (e.g., Nuske et al., 2014, 2015; Laeng et al., 2018; for a review, see de Vries et al., 2021). PLR, baseline, and task-related pupil measures have all been discussed in terms of ANS contributions (e.g., Bradley et al., 2008; Anderson and Colombo, 2009; Wang et al., 2016), but the latter two measures have also been the focus of research studying the locus coeruleus–norepinephrine (LC-NE) system, which is located in the brainstem and has roles in cognitive processes such as attention shifting and in regulating sensory processing and sympathetic activity (for a review see Steinhauer et al., 2004). More work is needed to better understand why autonomic activity and subcortical routes might relate to different traits and behaviors.

Recently, studies have examined individual differences in autistic traits in non-autistic populations, which is part of a broader autism phenotype (BAP) approach. The BAP generally refers to autistic characteristics that are seen in varying degrees across autistic individuals and their relatives, as well as non-autistic individuals (Pickles et al., 2000). Studies have examined associations between task-induced pupil responses and autistic traits in non-autistic children and adults (e.g., DiCriscio and Troiani, 2017; Turi et al., 2018; DiCriscio et al., 2019). For example, in a combined sample of autistic and non-autistic children, DiCriscio and Troiani (2017) found that smaller changes in pupil size during pupil adaptation to light were associated with more social-communicative difficulties. Additionally, adults with more autistic traits showed differential patterns of pupil response during visual perception tasks, such as increased dilation of the pupil (DiCriscio et al., 2019). Together, these studies show that pupillary autonomic markers in children and adults can also reflect individual differences that might relate to the BAP.

The objective of the present study was to expand on past BAP work to further investigate the relationship between parasympathetic activity, using PLR measures, and autistic traits in a non-autistic sample including both children and adults. Based on work with autistic individuals (e.g., Fan et al., 2009), it was hypothesized that increased autistic traits would be associated with reduced PNS activity (i.e., weaker and slower PLR responses). Additionally, the current study aimed to examine whether there are differences in pupil response patterns between children and adults. Work by Daluwatte et al. (2012) found weaker PLR responses in children younger than 8 years old, so it was anticipated that children will show weaker PLR responses than adults.

## Methods

### Participants

Participants included 65 non-autistic children (*M*_*age*_ = 6.20 years, *SD* = 2.68; Range: 2 to 12 years; 33 male, 32 female) and 77 non-autistic adults (*M*_*age*_ = 20.34, *SD* = 4.67; Range: 18 to 46 years; 44 male, 32 female, 1 transmale). Children were recruited through in-person recruitment events, targeted mailings, and emails to families in the New York City and New Jersey area. Adult participants were college students in an introductory psychology course who had the opportunity to participate for course credit. For adult participants, informed consent was completed prior to the study, and for children, caregivers completed informed consent. All procedures were approved by the Institutional Review Board of the College of Staten Island, City University of New York.

### Procedure

**FIGURE 1:**
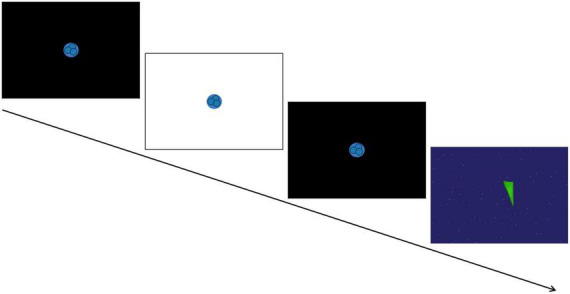
Schematic of the pupil light reflex (PLR) task. A trial consisted of a 120 ms white screen presented in between two black screens to induce the PLR response. This was followed by a brief video of moving shapes to avoid retinal saturation. [Paradigm adapted from Nyström et al. (2015)].

A SensoMotoric Instruments (SMI) RED eye-tracking system was used to measure gaze position and pupil size at 120Hz using iView software. Pupil diameter from both eyes was collected from an average distance of 65 cm from a 22″ widescreen monitor. A 5-point calibration sequence and 4-point validation was used at the start to confirm appropriate positioning and successful tracking. Following calibration, the PLR task began based on the stimuli used in Nyström et al. (2015). Each trial totaled 6 seconds and consisted of a fixation animation on a black screen that initially lasted either 1.6, 2, or 2.4 s (varying to avoid anticipatory pupil responses), then the screen flashed white for 120 ms while the fixation animation remained on the screen, and finally the black screen with the fixation animation resumed for the remainder of the trial. In between trials, an inter-trial video of moving shapes was presented for 10 s for children and for 15 s for adults to encourage saccades and prevent retinal saturation (see Figure 1 for schematic overview). Participants were instructed to look at the screen and attend to the PLR fixation animation until it disappeared from the screen. The experiment included nine trials, and each trial was initiated only after a clear indication that the participant was looking at the screen and the eye-tracker was successfully tracking their eye gaze. If the experimenter counted less than six potentially usable trials out of the initial nine (i.e., with attention allocated to the center of the screen before, during, and after the flash), the task was repeated and nine additional trials were presented.

### Assessment of autistic traits

Autistic traits were assessed using the Social Responsiveness Scale, Second Edition (SRS-2; Constantino and Gruber, 2012), a 65-item questionnaire measure designed to examine characteristics associated with autism that has been adopted by recent studies to examine variation in these traits in the general population (e.g., DiCriscio and Troiani, 2017). Adults completed the self-report Adult Form, and caregivers completed the Preschool Form (up to 4 years) or School-Age Form (4 years and older) about their child. T-scores were calculated for SRS-2 Total score, as well as for the Social Communication and Interaction (SCI) composite and RRB subscale. Higher SRS-2 scores are associated with increased levels of autistic traits.

### Data processing and analysis

Custom Python scripts were used to process the PLR dilation time series to identify PLR metrics for each trial. There were two initial inclusion criteria used for each eye for each trial: (1) no more than 100 ms of pupil data was missing during the first 1500 ms after the flash (e.g., due to blinks) and (2) valid pupil data was required at the time of the flash. Based on approaches taken in past PLR work (e.g., Fan et al., 2009; Nyström et al., 2015), pupil diameter for each eye for included trials was processed using a degree-2 Savitzky-Golay filter with a window of 11 samples to yield smoothed diameter and acceleration series, which were then further smoothed using a Gaussian convolution with a standard deviation of 5 samples. A final set of criteria were used to ensure that the resulting data accurately reflected the PLR curve: (1) the point of greatest minimum amplitude was reached within 1500 ms of the flash, (2) the point of greatest negative velocity was reached within 750 ms of the flash, and (3) the point of greatest negative acceleration was reached within 500 ms of the flash (see Supplementary Table 1 for number of eye trials excluded at each stage of processing for children and adults).

Based on past findings with infants, children, and adults, pupil measures calculated during the PLR task included (a) *baseline pupil diameter* (A_0_; e.g., Anderson and Colombo, 2009), (b) *relative constriction amplitude*, calculated from A_0_ and A_m_ (minimum diameter) as (A_0_^2^ - A_m_^2^)/A_0_^2^ (e.g., Fan et al., 2009), (c) *absolute constriction amplitude*, A_0_-A_m_ (e.g., DiCriscio and Troiani, 2017); and (d) *median constriction latency*, calculated as median latency to reach maximum negative acceleration (e.g., Nyström et al., 2015). Of these four measures, it should be noted that relative constriction amplitude and constriction latency have been most consistently found to reflect PNS activity (Wang et al., 2016). When clean data was available for both eyes on a given trial, PLR variables were averaged across both eyes, and then PLR metrics were averaged across usable trials for each participant.

PLR analyses focused on participants with four or more valid trials (*M*_trials_ = 7.08, *SD* = 1.73, range: 4-11; e.g., Nyström et al., 2015), leading to the exclusion of six children and eight adults. Three additional adults were excluded because they were age outliers (see Statistical analysis for more information). The final included sample therefore included 59 children (*M_*age*_* = 6.36 years, *SD* = 2.72 years, age range: 2-12 years) and 66 adults (*M_*age*_* = 19.64 years, *SD* = 2.04 years, age range: 18-28 years). For an illustration of the average PLR response over time for adults and children see Supplementary Figure 1, and for histograms illustrating distributions of the PLR measures see Supplementary Figure 2.

### Statistical analysis

The primary analyses included (1) a series of correlations to examine associations between PLR measures and autistic traits for each group, based on the SRS-2, and (2) a series of independent samples t-tests to examine developmental differences in the PLR between children and adults. Prior work across childhood (e.g., Daluwatte et al., 2012) and adulthood (e.g., Telek et al., 2018) has found age to be a significant factor in pupillary responses to light. Because of these past findings, and due to the wide age ranges for both groups, an age outlier check was conducted within each group. Participants who fell more than 3 SDs above or below the age mean were excluded from subsequent analyses. This resulted in the exclusion of three adult participants (aged 37 to 46 years; see Telek et al., 2018 for discussion of adult age-related differences).

With age outliers removed, a series of preliminary correlations were run to examine the relationship between age and PLR measures within each sample. Results showed that in children, age was positively associated with baseline pupil diameter (*r*(53) = 0.31, *p* = 0.020), suggesting that older children have greater pupil diameter at baseline. In adults, no associations were found between age and PLR measures (*p*s > 0.22). In subsequent correlational analyses, because age was associated with PLR measures in children, partial correlations controlling for age were used for the child sample, while standard bivariate correlations were used for adults.

## Results

### Correlational analyses

#### Relations among pupil measures

An initial set of correlations examined relations among the four pupil response measures, using a Bonferroni correction accounting for six comparisons for each age group (critical *p* = 0.05 / 6 = 0.0083). Analyses included partial correlations accounting for age for children, and bivariate correlations for adults. At both ages, several variables were significantly correlated with each other (see Supplementary Tables 2, 3 for correlation tables): PLR absolute constriction amplitude was positively associated with baseline pupil diameter and with PLR relative constriction amplitude (*p*s < 0.001). Further, in the adult sample only, PLR constriction latency was negatively associated with PLR relative constriction amplitude (*p* = 0.002). No other results held after the corrected p-value (see Supplementary Tables 2, 3).

#### Relations between autistic traits and pupil measures

The primary correlational analyses examined relations between autistic traits and pupil responses in children and adults, using a Bonferroni correction taking into account associations between SRS-2 scores and the four different pupil measures (critical *p* = 0.05 / 4 = 0.0125). For children, partial correlations were used, controlling for age, and for adults, bivariate correlations were used (see Supplementary Tables 4, 5 for the full results).

**Children.** After controlling for age, findings showed that RRB was negatively associated with baseline pupil diameter (*r*(50) = −0.32, *p* = 0.022), however, this finding did not survive the corrected *p*-value. Non-significant trends were also found that suggested greater absolute constriction amplitude was marginally related to lower levels of autistic traits overall, as well as SCI specifically (*r*s > −0.25, *p*s < 0.10; see Supplementary Table 4 for full results).

**Adults.** Bivariate correlations showed a significant negative correlation between SRS-2 Total score and relative constriction amplitude (*r*(64) = −0.28, *p* = 0.024), however this finding did not survive the corrected *p*-value. A non-significant trend was also found between SRS-2 Total and median latency (*r*(64) = 0.21, *p* = 0.092). No other PLR measures were significantly associated with overall level of autistic traits (*p*s > 0.40).

**FIGURE 2:**
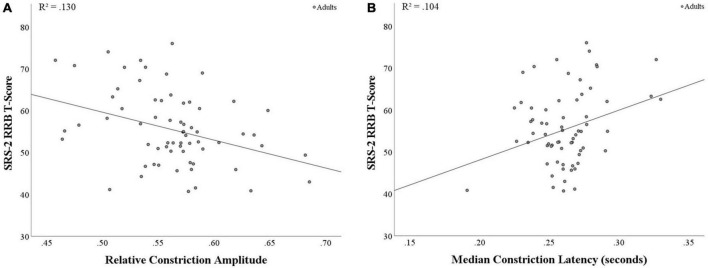
Correlations between PLR metrics and SRS-2 RRB score in adults. **(A)** A significant negative correlation was found between relative constriction amplitude and RRB (*p* = 0.003), with stronger PLR response associated with lower RRB scores. **(B)** A significant positive correlation was found between median latency and RRB (*p* = 0.008), with faster time to the point of maximum negative acceleration (i.e., shorter PLR response) associated with lower RRB scores.

When examining correlations between SRS-2 SCI and RRB scores in relation to pupil measures, RRB was found to be negatively associated with relative constriction amplitude (*r*(64) = −0.36, *p* = 0.003; see Figure 2A) and positively associated with median latency (*r*(64) = 0.32, *p* = 0.008; see Figure 2B), with both findings surviving the corrected *p*-value. This suggests that increased levels of RRB are associated with smaller relative pupil constriction and longer latency to respond to light, indicating weaker and slower PLR. Additionally, SRS-2 SCI and relative constriction amplitude were marginally associated (*r*(64) = −0.21, *p* = 0.088; see Supplementary Table 5 for full results).

### Group comparisons

A series of independent-samples t-tests examined differences in pupillary responses between children and adults. A Bonferroni correction was applied, taking into account group comparisons for the four different pupil measures (critical *p* = 0.05 / 4 = 0.0125).

**FIGURE 3:**
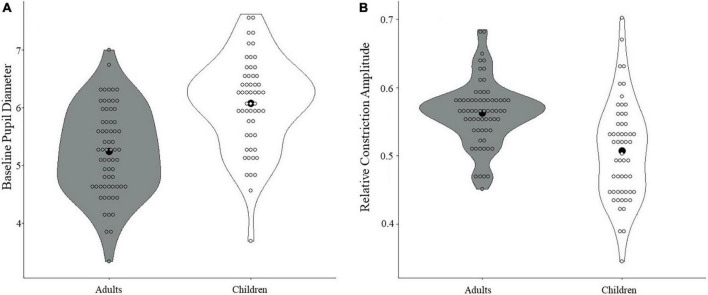
Differences in PLR measures between children and adults. **(A)** A significant difference in baseline pupil diameter was found, with smaller baseline pupil diameter in adults compared with children (*p* < 0.001). **(B)** A significant difference in relative constriction amplitude was found, with greater relative constriction amplitude in adults than in children (*p* < 0.001). Black dots denote the mean.

When comparing adults and children on the pupil measures, adults were found to have smaller baseline pupil diameter than children (*t*(119) = 5.88, *p* < 0.001, Cohen’s *d* = 1.07; see Figure 3A). Additionally, adults showed greater relative constriction amplitude than children (*t*(119) = 5.12, *p* < 0.001, Cohen’s *d* = 0.94; see Figure 3B), but no differences were found for median latency or absolute constriction amplitude (*p*s > 0.30; see Supplementary Figures 1, 2 for further data visualization). All results held with and without correction.

## Discussion

The current study used a PLR task adapted from Nyström et al. (2015) and had two main aims: first, to examine PLR responses in non-autistic children and adults in relation to levels of autistic traits, and second, to examine the differences in PLR responses across age groups. Main findings showed that (1) adults with increased levels of RRB showed a less robust PLR response (i.e., slower and weaker constriction), and (2) children showed larger pupil diameter at baseline and weaker relative PLR constriction compared with adults.

In relation to autistic traits, the current study showed that in children, after controlling for age, no relations between autistic traits and pupil measures survived correction for multiple comparison. However, trends were found showing that children who have increased levels of RRB also have smaller pupil diameter at baseline. Additionally, trends were found whereby higher levels of autistic traits overall and the SCI composite were both marginally correlated with a smaller absolute amplitude change during the PLR. Although this was not significant in the current sample, these trends align with findings from DiCriscio and Troiani (2017), showing that changes in pupil size during light adaptation were associated with differences in autistic traits in children, with significant results relating to the SRS-2 SCI composite score, but not the RRB subscale (DiCriscio and Troiani, 2017). Relatedly, in work with infants with and without an older autistic sibling, PLR relative constriction amplitude at 9 to 10 months predicted autistic traits at three years old, again, with significant findings focused on overall scores and social metrics, but not RRB (Nyström et al., 2018). More work is needed to understand why PLR metrics related to PNS responding might be more predictive of social-communication measures in children as compared to RRB, which might relate more to baseline pupil measures as suggested by the current work.

In adults, after controlling for multiple comparisons, overall levels of autistic traits showed a trend towards a negative association with relative constriction. When examining the subscales of autistic traits (SCI and RRB) in relation to PLR responses, significant findings after correction indicated that adults with increased levels of RRB showed both smaller relative constriction and longer latency to reach the point of constriction onset (point of maximum negative acceleration; e.g., Fan et al., 2009), indicating weaker and slower PLR on two well-studied markers of parasympathetic control. These findings suggest that, among a non-clinical sample of adults, those with better parasympathetic control endorsed fewer RRB. Although PLR measures have not been found to relate consistently to RRB in children, research in autistic and non-autistic children using cardiac autonomic measures has also found that increased respiratory sinus arrhythmia, a measure of better parasympathetic control, was related to lower levels of RRB across both groups (Condy et al., 2017). Taken together, these studies point to a role for parasympathetic markers in predicting adaptive functioning in the domain of RRB, but suggest that pupil measures and cardiac measures may be related to RRB at different points in development. It is important to note that the findings above in children showed no association between RRB and PNS-related PLR measures, in contrast to the findings with the adult sample. Further research is needed to clarify why different PLR measures were associated with autistic traits in adults but not in children, and to elucidate the mechanisms that might underlie these non-parallel results.

Examining past work linking sensory processing and RRB allows for a better understanding of the current associations between PLR and increased RRB in adults. Past research suggests that increased RRB are associated with difficulties in sensory processing in adults (Hwang et al., 2020; Moore et al., 2021) and children (Gabriels et al., 2008; Chen et al., 2009; Boyd et al., 2010; Schulz and Stevenson, 2019), and that smaller PLR constriction is related to more atypical sensory features in autistic children (Daluwatte et al., 2015). Increased sensitivity to sensory input, such as light, can lead to experienced overstimulation that might cause distress that needs to be regulated with the first available self-regulation method, such as RRB (e.g., Militerni et al., 2002; Baker et al., 2008). Because the PLR is a neurological measure of sensitivity to light (Beatty and Lucero-Wagoner, 2000; Lynch, 2018), increased RRB in relation to the PLR might imply elevated sensitivity to sensory stimuli in the environment. Results from the current study provide further support for the link between sensory sensitivity and RRB, as weaker PLR responses might indicate that the pupil diameter is not effectively and optimally regulating the amount of light that enters the eye, which can lead to more intense experiences with visual stimuli in the environment. With the PLR reflecting a key neural pathway in visual sensory processing, and with variation in sensory processing in autistic and non-autistic individuals relating to behavioral challenges, it could be posited that the relationship between sensory processing and some autism-related behaviors might be driven by this physiological mechanism.

In the present study, when examining overall developmental differences between children and adults, children showed increased pupil diameter at baseline in comparison to adults (for related work, see Telek et al., 2018). Additionally, children showed smaller relative constriction of the pupil in response to light, and this weaker PLR response in children also suggests weaker parasympathetic control. In a study that examined developmental trajectories of PLR responses in children and adolescents between 6 and 17 years of age, relative pupil constriction was found to increase (i.e., PLR became stronger) between the ages of 6 and 8 years, and then stabilized from ages 8 to 17 years (Daluwatte et al., 2012). In the current study, children and adults differed in relative constriction levels, but when looking at each age group separately, relative constriction was not associated with age. Although several studies have attempted to capture developmental changes in PLR metrics (Dinalankara et al., 2017; Telek et al., 2018), the specific developmental trajectory of PLR responses from infancy to adulthood in non-clinical populations is not yet clear, especially when examining it continuously. Future work should continue to examine trajectories of parasympathetic activity from infancy across development through changes in pupillary responding, exploring how these trends change across a wide age range.

The current study had several limitations. First, because the current sample did not include participants between the ages of 12 and 18 years, it was not possible to examine age as a continuous measure, limiting conclusions about the developmental trends in sensory responding seen through the PLR. This will be important to examine in future research, especially as hormonal changes associated with puberty might play a role in changes across age. A second limitation of the current study is that baseline pupil diameter was calculated during the PLR task, just before the flash occurred, and no baseline measurement outside the task was recorded. Future work should examine how differences in baseline calculation might affect age-related differences in pupillary measures, and how different baseline calculations might differ in relation to autistic traits.

Extending previous work that has found a less robust PLR response in autistic children (e.g., Fan et al., 2009) and a negative association between light adaptation responses and autistic traits in a broad population of children (DiCriscio and Troiani, 2017), the current study found that young adults with weaker and slower PLR have higher levels of RRB. Altogether, this points to the PLR as a marker associated with the broader autism phenotype, as opposed to an autism diagnosis. This well-studied marker of autonomic functioning could therefore provide an important window into the study of individual differences in adaptive behavior for both autistic and non-autistic individuals.

## Data availability statement

The de-identified data that support the findings of this study are available on request from the corresponding author JBW, jen.wagner@alum.mit.edu. The data are not publicly available due to privacy restrictions.

## Ethics statement

The present study involving human participants was reviewed and approved by the Institutional Review Board of the College of Staten Island, City University of New York (Protocol #570016). Adult participants provided written informed consent to participate in this study; for child participants, written informed consent was provided by the child’s legal guardian.

## Author contributions

SS-E contributed to the design of the study, data collection, organization, processing, statistical analysis, and wrote the initial draft of the current manuscript. AL contributed to data collection and the writing of portions of a previous version of the manuscript. SRS developed Python scripts for pupillometry data preparation and analysis. JBW contributed to the conception, design of the study and approach to analysis, and contributed to editing of all sections of the manuscript. All authors read and approved the final submitted version of the manuscript.

## References

[B1] Abbott A. Linke A. Nair A. Jahedi A. Alba L. Keown C. (2018). Repetitive behaviors in autism are linked to imbalance of corticostriatal connectivity: A functional connectivity MRI study. *Soc. Cogn. Affect. Neurosci.* 13 32–42. 10.1093/scan/nsx129 29177509 PMC5793718

[B2] American Psychiatric Association (2013). *Diagnostic and statistical manual of mental disorders fifth Edition.* Arlington: American Psychiatric Association, 991. 10.1176/appi.books.9780890425596

[B3] Anderson C. Colombo J. (2009). Larger tonic pupil size in young children with autism spectrum disorder. *Dev. Psychobiol.* 51 207–211. 10.1002/dev.20352 18988196 PMC3744086

[B4] Baker A. Lane A. Angley M. Young R. (2008). The relationship between sensory processing patterns and behavioural responsiveness in autistic disorder: A pilot study. *J. Autism Dev. Disord.* 38 867–875. 10.1007/s10803-007-0459-0 17899349

[B5] Bast N. Boxhoorn S. Supér H. Helfer B. Polzer L. Klein C. (2021). Atypical arousal regulation in children with autism but not with attention-deficit/hyperactivity disorder as indicated by pupillometric measures of locus coeruleus activity. *Biol. Psychiatry Cogn. Neurosci. Neuroimaging* S2451-9022, 117–118. 10.1016/j.bpsc.2021.04.010 33930603

[B6] Bast N. Poustka L. Freitag C. (2018). The locus coeruleus–norepinephrine system as pacemaker of attention – a developmental mechanism of derailed attentional function in autism spectrum disorder. *Eur. J. Neurosci.* 47 115–125. 10.1111/ejn.13795 29247487

[B7] Beatty J. Lucero-Wagoner B. (2000). “The pupillary system,” in *Handbook of psychophysiology*, eds Cacioppo J. T. Tassinary L. G. Berntson G. G. (Cambridge: Cambridge University Press), 142–162.

[B8] Blaser E. Eglington L. Carter A. Kaldy Z. (2014). Pupillometry reveals a mechanism for the autism spectrum disorder (asd) advantage in visual tasks. *Sci. Rep.* 4:4301. 10.1038/srep04301 24603348 PMC3945923

[B9] Bower M. Sweidan A. Xu J. Stern-Neze S. Yu W. Groysman L. (2021). Quantitative pupillometry in the intensive care unit. *J. Intens. Care Med.* 36 383–391. 10.1177/0885066619881124 31601157

[B10] Boyd B. Baranek G. Sideris J. Poe M. Watson L. Patten E. (2010). Sensory features and repetitive behaviors in children with autism and developmental delays. *Autism Res.* 3 78–87. 10.1002/aur.124 20437603 PMC3071028

[B11] Bradley M. Miccoli L. Escrig M. Lang P. (2008). The pupil as a measure of emotional arousal and autonomic activation. *Psychophysiology* 45 602–607. 10.1111/j.1469-8986.2008.00654.x 18282202 PMC3612940

[B12] Bremner F. (1999). in *The Pupil: Anatomy, physiology, and clinical applications*, Vol. 2001 ed. Loewenfeld E. (Oxford: Butterworth-Heinemann), 1881–1883. 10.1093/brain/124.9.1881

[B13] Cantor R. Navarro L. Won H. Walker R. Lowe J. Geschwind D. H. (2018). ASD restricted and repetitive behaviors associated at 17q21.33: Genes prioritized by expression in fetal brains. *Mol. Psychiatry* 23 993–1000. 10.1038/mp.2017.114 28533516 PMC5700871

[B14] Chen Y. Rodgers J. McConachie H. (2009). Restricted and repetitive behaviours, sensory processing and cognitive style in children with autism spectrum disorders. *J. Autism Dev. Disord.* 39 635–642. 10.1007/s10803-008-0663-6 19015969

[B15] Cocker K. Fielder A. Moseley M. Edwards A. (2005). Measurements of pupillary responses to light in term and preterm infants. *Neuro Ophthalmol.* 29 95–101. 10.1080/01658100590958274

[B16] Condy E. Scarpa A. Friedman B. (2017). Respiratory sinus arrhythmia predicts restricted repetitive behavior severity. *J. Autism Dev. Disord.* 47 2795–2804. 10.1007/s10803-017-3193-2 28616855

[B17] Constantino J. N. Gruber C. P. (2012). *Social responsiveness scale: SRS-2.* Torrance, CA: Western Psychological Services.

[B18] Daluwatte C. Miles J. Christ S. Beversdorf D. Lofgreen A. Berliner N. (2012). “Age-dependent pupillary light reflex parameters in children,” in *In Proceedinds of the 2012 Annual International Conference of the IEEE Engineering in Medicine and Biology Society*, (Piscataway), 3776–3779. 10.1109/EMBC.2012.6346789 23366750

[B19] Daluwatte C. Miles J. Christ S. Beversdorf D. Takahashi T. Yao G. (2013). Atypical pupillary light reflex and heart rate variability in children with autism spectrum disorder. *J. Autism Dev. Disord.* 43 1910–1925. 10.1007/s10803-012-1741-3 23248075 PMC3619026

[B20] Daluwatte C. Miles J. Sun J. Yao G. (2015). Association between pupillary light reflex and sensory behaviors in children with autism spectrum disorders. *Res. Dev. Disabil.* 37 209–215. 10.1016/j.ridd.2014.11.019 25528080 PMC4314503

[B21] de Vries L. Fouquaet I. Boets B. Naulaers G. Steyaert J. (2021). Autism spectrum disorder and pupillometry: A systematic review and meta-analysis. *Neurosci. Biobehav. Rev.* 120 479–508. 10.1016/j.neubiorev.2020.09.032 33172600

[B22] DiCriscio A. Troiani V. (2017). Pupil adaptation corresponds to quantitative measures of autism traits in children. *Sci. Rep.* 7:6476. 10.1038/s41598-017-06829-1 28743966 PMC5526922

[B23] DiCriscio A. Hu Y. Troiani V. (2019). Brief report: Pupillometry. Visual perception, and ASD Features in a task-switching paradigm. *J. Autism Dev. Disord.* 49 5086–5099. 10.1007/s10803-019-04213-8 31489540

[B24] Dinalankara D. Miles J. Nicole Takahashi T. Yao G. (2017). Atypical pupillary light reflex in 2–6-year-old children with autism spectrum disorders. *Autism Res.* 10 829–838. 10.1002/aur.1745 28188684

[B25] Eack S. Wojtalik J. Keshavan M. Minshew N. (2017). Social-cognitive brain function and connectivity during visual perspective-taking in autism and schizophrenia. *Schizophr. Res.* 183 102–109. 10.1016/j.schres.2017.03.009 28291690 PMC5432384

[B26] Ecker C. Pretzsch C. Bletsch A. Mann C. Schaefer T. Ambrosino S. (2022). Interindividual differences in cortical thickness and their genomic underpinnings in autism spectrum disorder. *Am. J. Psychiatry* 179 242–254. 10.1176/appi.ajp.2021.20050630 34503340

[B27] Falck-Ytter T. (2008). Face inversion effects in autism: A combined looking time and pupillometric study. *Autism Res. Off. J. Int. Soc. Autism Res.* 1 297–306. 10.1002/aur.45 19360681

[B28] Fan X. Miles J. Takahashi N. Yao G. (2009). Abnormal transient pupillary light reflex in individuals with autism spectrum disorders. *J. Autism Dev. Disord.* 39 1499–1508. 10.1007/s10803-009-0767-7 19499319

[B29] Gabriels R. Agnew J. Miller L. Gralla J. Pan Z. Goldson E. (2008). Is there a relationship between restricted, repetitive, stereotyped behaviors and interests and abnormal sensory response in children with autism spectrum disorders? *Res. Autism Spectr. Disord.* 2 660–670. 10.1016/j.rasd.2008.02.002

[B30] Goldwater B. (1972). Psychological significance of pupillary movements. *Psychol. Bull.* 77 340–355. 10.1037/h0032456 5021049

[B31] Hall C. Chilcott R. (2018). Eyeing up the future of the pupillary light reflex in neurodiagnostics. *Diagnostics* 8:19. 10.3390/diagnostics8010019 29534018 PMC5872002

[B32] Hwang Y. Arnold S. Srasuebkul P. Trollor J. (2020). Understanding anxiety in adults on the autism spectrum: An investigation of its relationship with intolerance of uncertainty, sensory sensitivities and repetitive behaviours. *Autism* 24 411–422. 10.1177/1362361319868907 31416327

[B33] Iaizzo P. Fitzgerald K. (2015). “Autonomic nervous system,” in *Handbook of cardiac anatomy, physiology, and devices [Internet]*, ed. Iaizzo P. (Cham: Springer International Publishing), 235–250. 10.1007/978-3-319-19464-6_14

[B34] Jung M. Tu Y. Lang C. Ortiz A. Park J. Jorgenson K. (2019). Decreased structural connectivity and resting-state brain activity in the lateral occipital cortex is associated with social communication deficits in boys with autism spectrum disorder. *NeuroImage* 190 205–212. 10.1016/j.neuroimage.2017.09.031 28927730

[B35] Laeng B. Færevaag F. Tanggaard S. von Tetzchner S. (2018). Pupillary responses to illusions of brightness in autism spectrum disorder. *Percept* 9:2041669518771716. 10.1177/2041669518771716 29796241 PMC5960863

[B36] Levy D. Rowley D. Abraham R. (1992). Portable infrared pupillometry using Pupilscan: Relation to somatic and autonomic nerve function in diabetes mellitus. *Clin. Auton. Res.* 2 335–341. 10.1007/BF01824304 1422101

[B37] Lynch G. (2018). Using pupillometry to assess the atypical pupillary light reflex and LC-NE System in ASD. *Behav. Sci.* 8:108. 10.3390/bs8110108 30469373 PMC6262612

[B38] Martineau J. Hernandez N. Hiebel L. Roché L. Metzger A. Bonnet-Brilhault F. (2011). Can pupil size and pupil responses during visual scanning contribute to the diagnosis of autism spectrum disorder in children? *J. Psychiatr. Res.* 45 1077–1082. 10.1016/j.jpsychires.2011.01.008 21679969

[B39] McKinnon C. Eggebrecht A. Todorov A. Wolff J. Elison J. Adams C. (2019). Restricted and repetitive behavior and brain functional connectivity in infants at risk for developing autism spectrum disorder. *Biol. Psychiatry Cogn. Neurosci. Neuroimaging* 4 50–61. 10.1016/j.bpsc.2018.09.008 30446435 PMC6557405

[B40] Militerni R. Bravaccio C. Falco C. Fico C. Palermo M. (2002). Repetitive behaviors in autistic disorder. *Eur. Child Adolesc. Psychiatry* 11 210–218. 10.1007/s00787-002-0279-x 12469238

[B41] Moore H. Brice S. Powell L. Ingham B. Freeston M. Parr J. (2021). The mediating effects of alexithymia, intolerance of uncertainty, and anxiety on the relationship between sensory processing differences and restricted and repetitive behaviours in autistic adults. *J. Autism Dev. Disord.* 52 4384–4396. 10.1007/s10803-021-05312-1 34643864 PMC9508023

[B42] Nuske H. Vivanti G. Dissanayake C. (2014). Brief report: Evidence for normative resting-state physiology in autism. *J. Autism Dev. Disord.* 44 2057–2063. 10.1007/s10803-014-2068-z 24550080

[B43] Nuske H. Vivanti G. Dissanayake C. (2015). No evidence of emotional dysregulation or aversion to mutual gaze in preschoolers with autism spectrum disorder: An eye-tracking pupillometry study. *J. Autism Dev. Disord.* 45 3433–3445. 10.1007/s10803-015-2479-5 26031923

[B44] Nyström P. Gliga T. Jobs E. Gredebäck G. Charman T. Johnson M. (2018). Enhanced pupillary light reflex in infancy is associated with autism diagnosis in toddlerhood. *Nat. Commun.* 9 1–5. 10.1038/s41467-018-03985-4 29735992 PMC5938234

[B45] Nyström P. Gredebäck G. Bölte S. Falck-Ytter T. (2015). Hypersensitive pupillary light reflex in infants at risk for autism. *Mol. Autism* 6 1–6. 10.1186/s13229-015-0011-6 25750705 PMC4352563

[B46] Pickles A. Starr E. Kazak S. Bolton P. Papanikolaou K. Bailey A. (2000). Variable expression of the autism broader phenotype: Findings from extended pedigrees. *J. Child Psychol. Psychiatry* 41 491–502. 10.1111/1469-7610.00634 10836679

[B47] Poe G. Foote S. Eschenko O. Johansen J. Bouret S. Aston-Jones G. (2020). Locus coeruleus: A new look at the blue spot. *Nat. Rev. Neurosci.* 21 644–659. 10.1038/s41583-020-0360-9 32943779 PMC8991985

[B48] Polzer L. Freitag C. Bast N. (2022). Pupillometric measures of altered stimulus-evoked locus coeruleus-norepinephrine activity explain attenuated social attention in preschoolers with autism spectrum disorder. *Autism Res.* 15 2167–2180. 10.1002/aur.2818 36111843

[B49] Ramaswami G. Geschwind D. (2018). *Genetics of autism spectrum disorder. In: Handbook of Clinical Neurology.* Amsterdam: Elsevier B.V, 321–329. 10.1016/B978-0-444-63233-3.00021-X 29325621

[B50] Sato W. Uono S. (2019). The atypical social brain network in autism: Advances in structural and functional MRI studies. *Curr. Opin. Neurol.* 32 617–621. 10.1097/WCO.0000000000000713 31135458

[B51] Schulz S. Stevenson R. (2019). Sensory hypersensitivity predicts repetitive behaviours in autistic and typically-developing children. *Autism* 23 1028–1041. 10.1177/1362361318774559 30244585

[B52] Steinhauer S. Siegle G. Condray R. Pless M. (2004). Sympathetic and parasympathetic innervation of pupillary dilation during sustained processing. *Int. J. Psychophysiol.* 52 77–86. 10.1016/j.ijpsycho.2003.12.005 15003374

[B53] Telek H. Erdol H. Turk A. (2018). The effects of age on pupil diameter at different light amplitudes. *Beyoglu Eye J.* 3 80–85. 10.14744/bej.2018.43534

[B54] Turi M. Burr D. Binda P. (2018). Pupillometry reveals perceptual differences that are tightly linked to autistic traits in typical adults. *eLife* 7:e32399. 10.7554/eLife.32399 29506652 PMC5839694

[B55] Wang C. Munoz D. P. (2015). A circuit for pupil orienting responses: Implications for cognitive modulation of pupil size. *Curr. Opin. Neurobiol.* 33 134–140. 10.1016/j.conb.2015.03.018 25863645

[B56] Wang Y. Zekveld A. Naylor G. Ohlenforst B. Jansma E. Lorens A. (2016). Parasympathetic nervous system dysfunction, as identified by pupil light reflex, and its possible connection to hearing impairment. *PLoS One* 11:e0153566. 10.1371/journal.pone.0153566 27089436 PMC4835104

[B57] Warrier V. Zhang X. Reed P. Havdahl A. Moore T. Cliquet F. (2022). Genetic correlates of phenotypic heterogeneity in autism. *Nat. Genet.* 54 1293–1304. 10.1038/s41588-022-01072-5 35654973 PMC9470531

[B58] Waye M. Cheng H. (2018). Genetics and epigenetics of autism: A Review. *Psychiatry Clin. Neurosci.* 72 228–244. 10.1111/pcn.12606 28941239

[B59] Wiśniowiecka-Kowalnik B. Nowakowska B. (2019). Genetics and epigenetics of autism spectrum disorder—current evidence in the field. *J. Appl. Genet.* 60 37–47. 10.1007/s13353-018-00480-w 30627967 PMC6373410

[B60] Wolff J. Swanson M. Elison J. Gerig G. Pruett J. Styner M. (2017). Neural circuitry at age 6 months associated with later repetitive behavior and sensory responsiveness in autism. *Mol. Autism* 8 1–12. 28316772 10.1186/s13229-017-0126-z PMC5351210

[B61] Yousaf A. Waltes R. Haslinger D. Klauck S. Duketis E. Sachse M. (2020). Quantitative genome-wide association study of six phenotypic subdomains identifies novel genome-wide significant variants in autism spectrum disorder. *Transl. Psychiatry* 10 1–11. 10.1038/s41398-020-00906-2 32624584 PMC7335742

