# Supplementary material for: Associations between the pupil light reflex and the broader autism phenotype in children and adults
Source: Front Hum Neurosci. 2023 Feb 21;16:1052604. doi: 10.3389/fnhum.2022.1052604 (PMC9990758; doi:10.3389/fnhum.2022.1052604)
Supplement: Supplementary file 1 [file Data_Sheet_1.pdf]

Supplementary Materials

Supplementary Table 1

Pupil Light Reflex Eye Trial Exclusions

	Children	Adults
More Than 100 ms Missing Data in First 1500 ms After Flash	414	260
No Valid Pupil Data at Time of Flash	22	20
Minimum Amplitude Not Reached within 1500 ms After Flash	5	9
Minimum Negative Velocity Not Reached within 750 ms After Flash	4	4
Minimum Negative Acceleration Not Reached within 500 ms After Flash	43	37

Note: Number of eye trials (left/right) excluded for each criterion; Total number of eye trials included: n = 874 for children; n = 1072 for adults

Supplementary Table 2

Partial Correlations Between Pupil Light Reflex Measures in Children After Controlling for Age

	Baseline Pupil Diameter	Relative Constriction Amplitude	Absolute Constriction Amplitude	Median Latency
Baseline Pupil Diameter	—	-.15 (.30)	.49* (<.001)	-.05 (.70)
Relative Constriction Amplitude	—	—	.78* (<.001)	-.29^ (.03)
Absolute Constriction Amplitude	—	—	—	-.28^ (.04)

Note: Partial correlations (r) shown with p-value in parentheses. ^ $p < .05$; * $p < .0083$

Supplementary Table 3

Correlations Between Pupil Light Reflex Measures and Autistic Traits in Adults

	Baseline Pupil Diameter	Relative Constriction Amplitude	Absolute Constriction Amplitude	Median Latency
Baseline Pupil Diameter	—	.03 (.84)	.81* (<.001)	-.11 (.40)
Relative Constriction Amplitude	—	—	.60* (<.001)	-.38* (.002)
Absolute Constriction Amplitude	—	—	—	-.32ˆ (.009)

Note : Correlations (*r*) shown with p-value in parentheses.ˆ $p < .05$; * $p < .0083$

Supplementary Table 4

Partial Correlations Between Pupil Light Reflex Measures and Autistic Traits in Children After Controlling for Age

	Baseline Pupil Diameter	Relative Constriction Amplitude	Absolute Constriction Amplitude	Median Latency
SRS-2 Total	-.22 (.12)	-.11 (.42)	-.23 (.09)	.04 (.80)
SRS-2 RRB	-.32ˆ (.022)	.01 (.93)	-.17 (.23)	-.12 (.40)
SRS-2 SCI	-.18 (.21)	-.15 (.28)	-.24 (.08)	-01 (.95)

Note: SRS-2 = Social Responsiveness Scale, Second Edition; RRB = restricted and repetitive behaviors; SCI = Social Communication and Interaction. Partial correlations (*r*) shown with p-value in parentheses. ˆ $p < .05$; * $p < .0125$

Supplementary Table 5

Correlations Between Pupil Light Reflex Measures and Autistic Traits in Adults

	Baseline Pupil Diameter	Relative Constriction Amplitude	Absolute Constriction Amplitude	Median Latency
SRS-2 Total	.11 (.40)	-.28ˆ (.024)	-.09 (.49)	.21 (.09)
SRS-2 RRB	.09 (.50)	-.36* (.003)	-.15 (.24)	.32* (.008)
SRS-2 SCI	.10 (.43)	-.21 (.09)	-.05 (.68)	.15 (.23)

Note: SRS-2 = Social Responsiveness Scale, Second Edition; RRB = restricted and repetitive behaviors; SCI = Social Communication and Interaction. Correlations (r) shown with p-value in parentheses. ˆ $p < .05$; * $p < .0125$

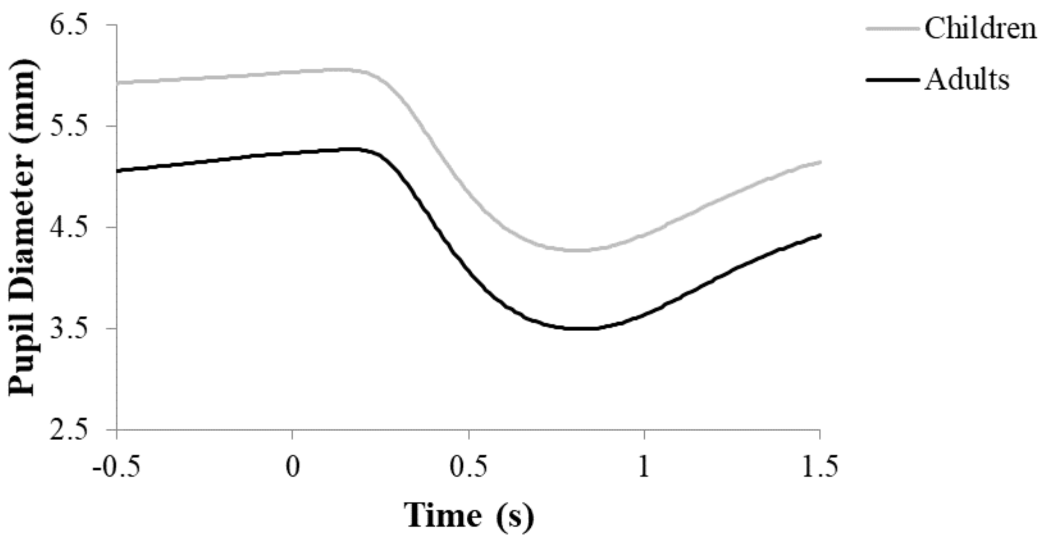

Supplementary Figure 1. Illustration of the PLR response over time averaged across children (gray line) and adults (black line). The zero time point denotes flash onset.

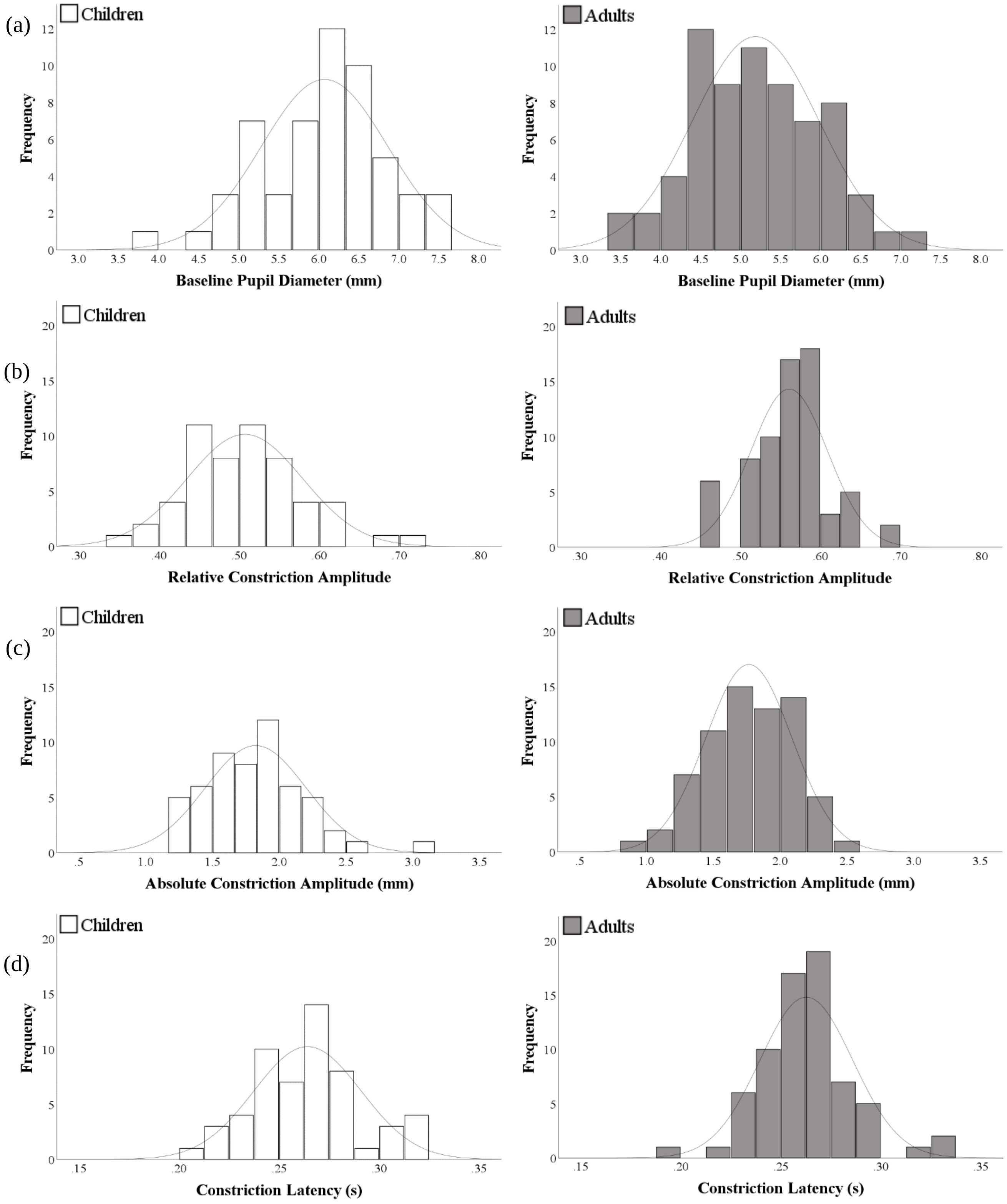

Supplementary Figure 2. Histograms showing the distribution of a) baseline pupil diameter (mm), b) relative constriction amplitude, c) absolute constriction amplitude (mm), and d) constriction latency in seconds (i.e., median latency) in children (left) and adults (right) samples.
